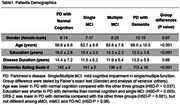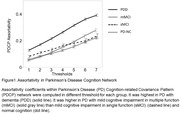# Cognition‐related Network Organization in Parkinson's Disease with Mild Cognitive Impairment and Dementia

**DOI:** 10.1002/alz.084270

**Published:** 2025-01-09

**Authors:** Yoshikazu Nakano, An Vo, Nha Nguyen, Chris C Tang, David Eidelberg

**Affiliations:** ^1^ The Feinstein Institutes for Medical Research, Manhasset, NY USA; ^2^ Albert Einstein College of Medicine, Bronx, NY USA

## Abstract

**Background:**

The Parkinson's disease (PD) cognition‐related covariance pattern (PDCP) was derived from network analysis of metabolic positron emission tomography (PET). The expression score is a feasible imaging biomarker that correlates with neuropsychological test performance. Graph analysis within specific networks characterizes brain function, particularly in terms of assortativity, which reflects the tendency to connect to nodes with similar degree values. However, PDCP assortativity is unclear.

**Methods:**

A total of 102 patients with PD underwent neuropsychological testing and [^18^F]‐fluorodeoxyglucose PET. Patients scoring less than 130 on the Dementia Rating Scale‐2 (DRS‐2) were classified as having PD with dementia (PDD). PD with mild cognitive impairment in single or multiple functions (sMCI or mMCI) was defined as having one (sMCI) or two or more (mMCI) of the following criteria: DRS‐2 subscore (Attention < 35, Initiation/Perseveration < 35, Construction < 6, Conceptualization < 35, Memory < 24), Wisconsin Card Sorting Test < 13 (executive), Hooper Visual Organization Test < 30 (visuospatial), and Boston Naming Test < 48 (language). The others were defined as having normal cognition (PD‐NC). We identified 35 anatomical regions of interest (ROIs) as nodes corresponding to the PDCP network. The pairwise correlation at each node of normalized metabolic activity derived from FDG‐PET data was calculated in each group by 100 bootstrapping iterations. The assortativity coefficient was calculated as the Pearson correlation coefficient of degrees between connected node pairs. Group differences were tested by repeated measures analysis of variance as group and threshold coefficients.

**Results:**

There were 22 PD‐NC, 24 sMCI, 31 mMCI, and 25 PDD. Demographic data are shown in Table 1. PDCP assortativity showed no significant differences between PD‐NC and sMCI (P = 0.23). However, it was increased in mMCI and PDD compared to PD‐NC and sMCI (P < 0.01). In addition, it was higher in PDD than in mMCI (P < 0.001) (Figure 1).

**Conclusions:**

Assortativity in the PDCP network is a potential predictor of the transition from MCI to dementia in PD patients. Furthermore, patients with cognitive impairment in multiple functions are at risk of developing dementia.